# The Influence of Step Load Periodisation Based on Time Under Tension in Hypoxic Conditions on Hormone Concentrations and Postoperative ACL Rehabilitation of a Judo Athlete: A Case Study

**DOI:** 10.3390/jcm14082549

**Published:** 2025-04-08

**Authors:** Miłosz Drozd, Wojciech Luboń, Jose Antonio Perez Turpin, Wojciech Grzyb

**Affiliations:** 1 Institute of Sport Sciences, The Jerzy Kukuczka Academy of Physical Education, 40-065 Katowice, Poland; wlubon@sum.edu.pl; 2 Department of Ophthalmology, Faculty of Medical Sciences, Medical University of Silesia, 40-752 Katowice, Poland; 3 Institute of I.U. Tourist Research, Department of General Didactic and Specific Didactic, University of Alicante, 03690 Alicante, Spain; jose.perez@gcloud.ua.es; 4 Faculty of Physical Education, Gdansk University of Physical Education and Sport, 80-336 Gdansk, Poland; wojciech.grzyb@awf.gda.pl

**Keywords:** growth hormone, insulin-like growth factor 1, erythropoietin, time under tension, tempo, periodisation, hypoxia, ACL rehabilitation, step load, VO_2_ max, isokinetic

## Abstract

The aim of this study was to determine the effect of a step load periodisation protocol for the rehabilitation of the anterior cruciate ligament (ACL) based on the variables of both the tempo of movement and time under tension (TUT) in normobaric hypoxia using a case study. **Introduction**: We verified the influence of variables such as time under tension (TUT) and the tempo of movement in hypoxia on the concentration of insulin-like growth factor 1 (IGF-1), growth hormone (GH), and erythropoietin (EPO). The effectiveness of the protocol also concerned variables such as peak torque of the knee flexors and extensors and maximum oxygen uptake (VO_2_max), as well as body composition analysis. **Methods:** The study used a 28-year-old judoka athlete from the national team, competing in the weight category up to 73 kg. **Results:** The use of short partial rest breaks between series (80s) in combination with six exercises in four series and a hypoxic environment (FiO_2_ = 15%) significantly increased metabolic stress, resulting in the highest increase in GH and IGF in the main phase of accumulation of the 3:1 step load. During 16 running sessions, the rehabilitated athlete achieved a significant increase in individual variables in the running test. **Conclusions:** The combination of a hypoxic environment combined with a periodized rehabilitation protocol can induce a number of positive hormonal, circulatory and respiratory reactions as well as positively influence muscle asymmetry, which can ultimately shorten the time it takes for an athlete to return to sport (RTS).

## 1. ACL in Judo

Judo is now the most widely practised martial art in the world [1]. Judo is a functional, long-range intermittent combat sport that requires application and tactical functionality to achieve success. It is an Olympic sport in which the key lies in the use of equipment, available and technical [2,3]. Judo involves at least four technical aspects: throw, hold, choke, and arm lock, each of which could put a lot of pressure on various people’s anatomical structures. For a throw to be effective, a judoka must manipulate the opponent’s center of gravity in relation to their base of support.

### 1.1. ACL in Judo

Taking into account the information contained in the above paragraph on the nature of the discipline, attention should be paid to the traumatic nature of the discipline. In total, 79.0% of the judokas have suffered injuries lasting more than three weeks, the most serious injury being an anterior cruciate ligament injury [4,5]. This injury led to the exclusion of players for a period of 3–12 weeks in 10% of the players, 3–6 months in 26%, 6–9 months in 32%, 9–12 months in 18%, and over 18% in 14% of judo competitors [5]. Another study found that injuries can occur anywhere in the body in judo, but the risk of injury to the knee is greater than any other anatomical structure, accounting for 20% of all judo injuries [6]. This was also shown in the study by [7], in which 133 of 145 first-year college judokas had experienced a knee injury in the past; 94 of them also reported numerous episodes of knee injury during a pre-season physical examination. The anterior cruciate ligament (ACL) injuries are the most common knee ligament injuries in injured judo athletes, in addition to medial collateral ligament injuries [8]. Likewise, an ACL injury is the most severe injury type reported for judo athletes [5]. The number of indirect ACL injuries is similar to the number of direct ACL injuries in this group; however, more data are needed for future studies examining indirect ACL injuries. Research by [9] showed that the majority of ACL injuries in both male and female judo athletes were contact injuries, and the ratio of direct and indirect ACL injuries was similar. Most ACL contact injuries occurred when an athlete was attacked by another judo competitor using the skills known as “Osotogari” and “Kosoto-gari”. The impact of ACL injuries on judo athletes is becoming quite problematic as it constitutes as much as 20–56% of all documented injuries in judo athletes [10].

### 1.2. Return to Sport

Considering that the process of returning to sport (RTS) after an injury can take up to several months, we are increasingly seeing the use of new forms of training methods aimed at maximizing the RTS stage, especially in competitive athletes. However, it is necessary to refer to the studies that divided the judokas after the entire RTS process of the anterior cruciate ligament that returned to the same level of sports. This work showed that only 32.0% of judokas reached the same level, 39% slightly decreased, 24.0% significantly decreased, and 5.0% gave up competitive training and participation in judo competitions [5]. Therefore, taking into account the maximisation of performance results and the constantly changing rules of competition in judo, as well as the relatively low percentage of returning to the same level of sport, we decided to use artificial high mountain conditions in our work based on the use of RTS periodisation based on the load progression method. One of the studies indicates that resistance exercises performed in a normobaric and hypoxic environment cause a number of structural and functional changes in skeletal muscles [11]. Many authors note that the main mechanisms responsible for this effect are related to an increased accumulation of metabolites due to hypoxia. Anaerobic exercise in hypoxia has been found to have a more favourable effect on metabolic cost and motor unit recruitment. Moreover, the use of moderate-intensity endurance training in hypoxia increases metabolic stress mechanisms resulting from physical exercise (cytokines, anabolic hormones, reactive oxygen species and oxidative stress factors), which are an important factor influencing muscle hypertrophy [12,13,14].

It is difficult to find data in contemporary literature describing and comparing the effectiveness of different resistance training strategies in normobaric hypoxia based on the use of periodic load progression in the ACL rehabilitation process. Creating rehabilitation protocols based on the periodisation of the training plan is a key element that determines the optimization of a specific micro- and mesocycle. This allows for controlled and targeted control of the process depending on its stage, where variables such as movement tempo, time under tension (TUT), the number of sets, and rest period between sets and exercises, in combination with the structure of the exercise, enable the occurrence of physiological adaptations [15,16,17].

### 1.3. The Rehabilitation Process

Keep in mind that complete ACL damage is often associated with long absences from training and competitions. The time it takes to return to full fitness is usually 9–12 months after anterior cruciate ligament reconstruction. It will depend on many factors, in particular the individual predispositions of the body, the athlete’s level of fitness before the injury, as well as involvement in the therapeutic process. There are also trends in recent decades that have moved towards accelerated programmes, often resulting in return to play (RTP) within 4–6 months after surgery; however, longer rehabilitation cycles have recently experienced a renaissance due to an improved understanding of graft healing and reconstruction schedules [18]. It is important to remember that the biological remodelling phase after ACL transplantation may be incomplete even 1 year after surgery [19]. The long postoperative recovery period encourages researchers to continually expand their knowledge of epidemiology. The paramount value is not only the return of the player to competitions, but in particular, the prevention of re-injury. Rehabilitation protocols include therapeutic and training processes that allow recovery. The constant search for new methods and means related to the most effective rehabilitation process encourages researchers to look for new methods to improve this process.

### 1.4. Hypoxic Training

Hypoxic training has been extensively studied in terms of its effect on circulatory and respiratory changes. Because this environment triggers a physiological response that affects the body’s performance by inducing tissue hypoxia through training under conditions of a lower partial pressure of oxygen (PO2), it a causes deterioration in athletes’ performance [20,21]. On the other hand, staying at altitude induces a number of adaptive changes that improve the body’s performance [22]. During hypoxia, the body produces hypoxia-inducible factor 1-α (HIF-1-α), causing the formation of new blood vessels, vascularization, and glycolysis. Taking into account our research problem, HIF-1α is activated during injuries, e.g., during sports. This factor increases blood flow by dilating blood vessels, increasing the partial pressure of oxygen in the blood, and inducing the synthesis of proteoglycans and fibronectin, as well as the production of collagen [23]. This shows that the hypoxic environment can have a beneficial effect on the process of tissue remodelling and on muscle hypertrophy and strength. The use of a hypoxic environment in our rehabilitation protocol RTS is important in terms of its effect on hormone levels and their effect on muscle hypertrophy and tissue remodelling. From the analysis of the literature review on the effect of the hypoxic environment on the concentration of hormones such as growth hormone (GH) or insulin-like growth factor 1 (IGF-1), we receive inconsistent research results [11,24,25,26,27].

Although modern surgical techniques allow for minimally invasive ACL reconstruction, postoperative muscle arthrosis is a concern in RTS, as the arthrosis itself can prolong safe return to previous levels of competition and contribute to the risk of reinjury [28]. Therefore, bear in mind that GH supplementation is banned by the World Anti-Doping Agency, and in collegiate and professional sports. Know also that increased levels of GH in the body helps cells and tissues grow and regenerate [28].

We decided to investigate the effect of RTS in a hypoxic environment on the process of tissue remodelling and on the hypertrophy and strength of the lower limbs and aerobic capacity. Therefore, the objective of this study was to examine the effectiveness of the practical use of high-altitude conditions in the rehabilitation protocol after arthroscopic reconstruction of the anterior cruciate ligament.

## 2. Materials and Methods

### 2.1. Surgical Procedure

#### 2.1.1. Diagnosis

Sprain, twist, and strain of the knee joint and knee ligaments or sprain and twist of other unspecified parts of the knee.

Consequences of other external causes: Consequences of an indeterminate external cause.

Right knee sprain. Anterior rotational instability. Complete damage to the anterior cruciate ligament. Damage to the posterior horn of the medial meniscus. Damage to the articular cartilage of the lateral femoral condyle of the fourth degree size of 5mm × 20 mm in the loading zone. subchondral fracture of the posterior part of the lateral condyle of the tibia with damage to the articular cartilage of the fourth degree of 5mm × 5 mm.

#### 2.1.2. Treatment

An arthroscopic reconstruction of the anterior cruciate ligament of the right knee joint was performed with a quadruple complex ST and GR muscle tendon graft.

Graft thickness 9.0 mm.

Attached to the thigh: TightRope II RT Arthrex (Arthrex Polska, Warsaw, Poland).

Attached to the shin: TightRope II ABS plus TightRope (Arthrex Polska, Warsaw, Poland) ABS button 20 mm.

Femoral tenodesis from the middle ⅓ of the iliotibial band.

Attached to the thigh: Swive Lock 4.75 Arthrex (Arthrex Polska, Warsaw, Poland).

The suturing of the posterior horn of the meniscus involved using 1× suture ale inside FastFix 360 (Arthrex Polska, Warsaw, Poland). Free fragments of articular cartilage were removed. The medial femoral condyle was cleaned.

### 2.2. Participant

The study used a 28-year-old judoka athlete from the national team, competing in the weight category up to 73 kg. Subjects entered the rehabilitation protocol under normobaric hypoxia 8 weeks after the arthroscopic reconstruction of the anterior cruciate ligament due to unilateral, posttraumatic, isolated anterior instability of the knee joint. The graft for the procedure was taken from the tendons of the hamstring muscles. The subject was informed about the research protocol and the resulting risks and benefits and then gave his written consent to participate in the research. In addition, competitors had the right to withdraw from participation in the tests at any time during their duration. Resignation could also occur at the request of the attending physician. The research protocol was approved by the Bioethics Committee for Scientific Research at The Academy of Physical Education in Katowice, Poland (No. 1/I/2021), and met the ethical standards of the Declaration of Helsinki.

### 2.3. An Experimental Approach to the Problem

The pilot study was conducted at two facilities. The rehabilitation protocol was carried out in the hypoxia laboratory at the Academy of Physical Education in Katowice, while diagnostics took place at the medical facility of Galen Rehabilitacja Sp. z o. o. (Bieruń, Poland).

**Figure 1:**
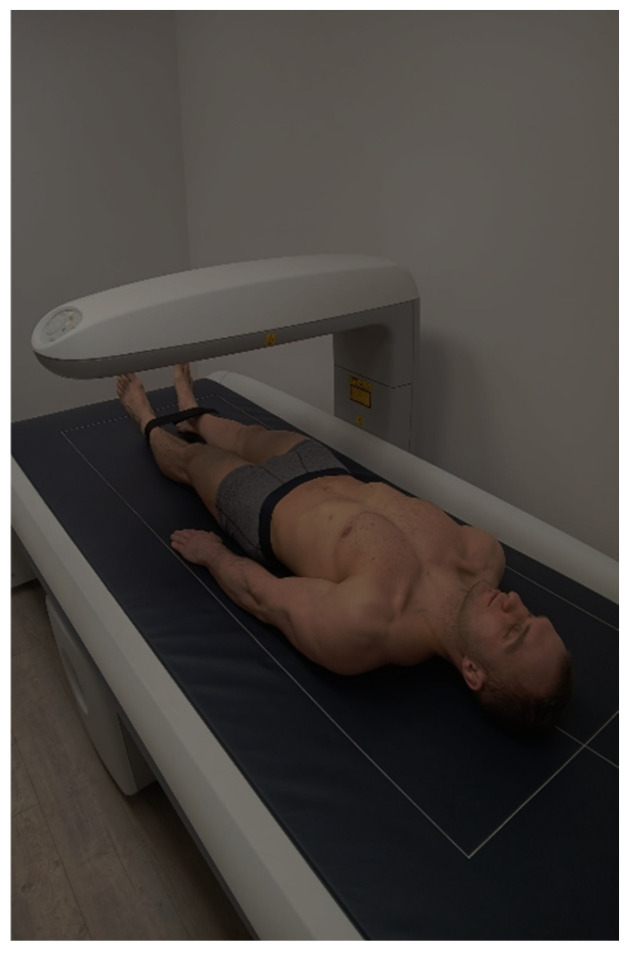
Densitometry photo of a judo athlete.

At 24 h before the test, the subject underwent morphological and hormonal tests, where resting GH and IGF concentrations were determined. A 6 mL blood sample was taken from the antrop fossa of the mediolateral vein to determine the concentration of GH/IGF-1 and erythropoietin (EPO). Blood collection was performed by qualified persons in accordance with occupational health and safety regulations. The collected blood was directly transported to the diagnostic laboratory. Another blood collection was performed immediately after the intervention (30 min after the last training session) at the end of the second and fourth mesocycle, corresponding to each microcycle (Figure 1). Additionally, the last collection was performed 48 h after the last training session.

Hormone concentration: GH was assessed in the serum using the Beckman Coulter IV D IRMA GH Ref. IM 1397 kit (GH, (µg/L) mlU/L), using the immunoradiometric method (ImmunoTech limited liability company, Praha, Czech Republic). Irisin concentration (IR, µg/mL; 0.2–2 µg/mL) was determined by the ELISA system (kit) BioVentor-Laboratorium medicina as Irisin ELISA Cat. No RAG018R, Praha, Czech Republic. IGF-1 (IGF-1α, ng/mL) was measured using the Beckman Coulter limited liability company, Warsaw, Poland, IV D IRMA IGF-1 kit Ref. A15729 ImmunoTech limited liability company, Praha, Czech Republic. Erythropoietin (EPO) was measured with the Sandwich ELISA system (kit) from BioVentor-Laboratorium medicina. The calibration range was 1.6–100 (mlU/mL), and the limit of detection was 0.14 (mIU/mL).

Bone mineral density was assessed using the densitometry (DXA) method. This was used to analyse body composition (the composition and distribution of fat tissue and muscle mass). Body composition analysis was performed under standardized conditions, i.e., in the morning (from 8 to 9 a.m.), 72 h before the study. The final measurement took place 48 h after the last 4 mesocycles.

**Figure 2:**
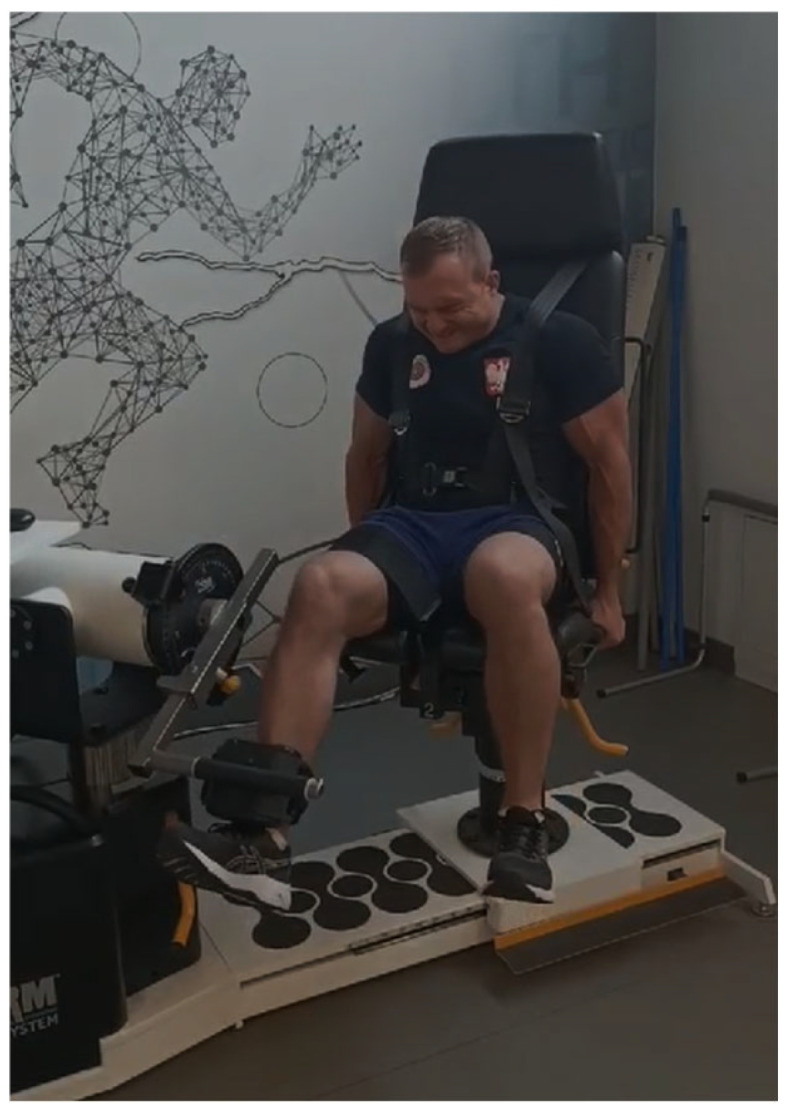
Photo taken during the test on an isokinetic dynamometer.

After consultation with a physician and considering the short postoperative period (8 weeks), the assessment of peak torque on the HUMAC NORM isokinetic dynamometer (Stoughton, MA, USA) was omitted. The baseline measurement of the peak torque of knee extensors and flexors was performed 12 weeks after the procedure and 48 h after the 4th mesocycle (Figure 2). Individual muscle groups activated during concentric contraction under isokinetic (constant) load in clinical conditions were assessed. The device was calibrated according to the instructions [29]. Immediately before the test, the athlete performed a warm-up (10 min/stationary bike/70–80 rpm). Adaptation to the isokinetic dynamometer began with the performance of 3 test repetitions (familiarization with the device). Additionally, before the main test attempt, the athlete performed 3 submaximal and two maximal repetitions. There was a 30 s rest break between repetitions, and a 3 min rest break between sets [30]. The subject was instructed to generate as much strength and power as possible during the main test. The display was positioned to allow the subject to receive real-time feedback. The subject sat in an upright position with a backrest at an angle of 85°U. The axis of rotation of the knee joint was aligned with the axis of rotation of the dynamometer. The lever arm pad was fixed at the head of the fibula so that movement of the ankle joint was not restricted.

Knee extensors and flexors were assessed at angular velocities:

- 60°/s^−1^ verification repetition and 5 test repetitions;
- 120°/s^−1^ verification repetition and 5 test repetitions;
- 180°/s^−1^ verification repetition and 15 test repetitions.

The range of joint mobility was determined by the extended flexion limit that an athlete can perform.

#### VO_2_max (Maximum Oxygen Uptake)

**Figure 3:**
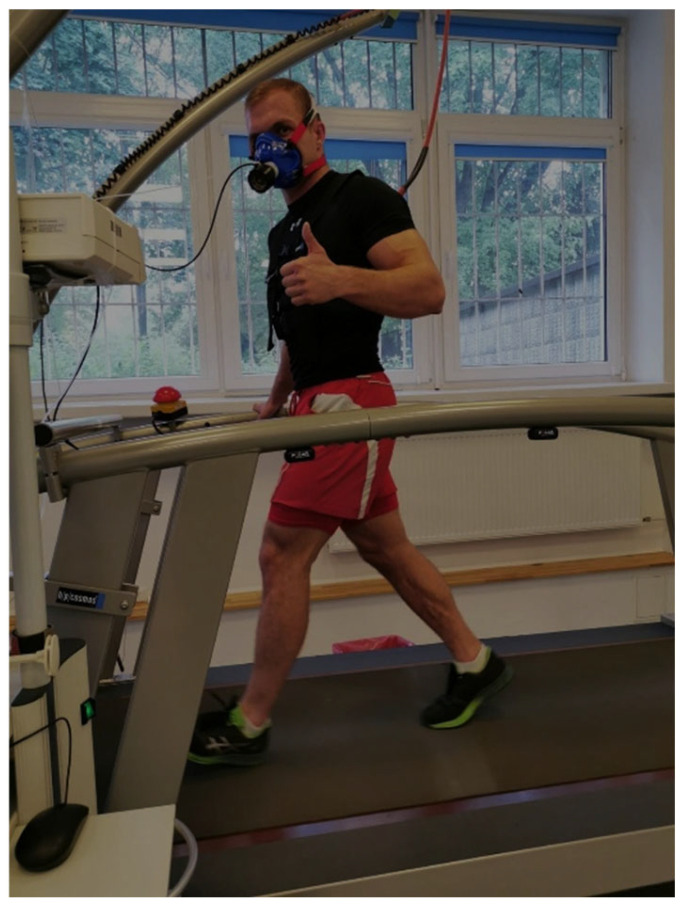
Photo during the VO_2_max test.

Because of the short postoperative period (8 weeks), the maximum oxygen uptake assessment (baseline measurements) was performed after consultation with a doctor in the 12th week after the procedure and 48 h after the 4th mesocycle (Figure 3). The VO_2_max was measured objectively and reliably in the laboratory by a direct analysis of gases associated with lung ventilation, using a modified Bruce protocol to perform a progressive treadmill test (Table 1) [31].

### 2.4. Resistance Training

**Figure 4:**
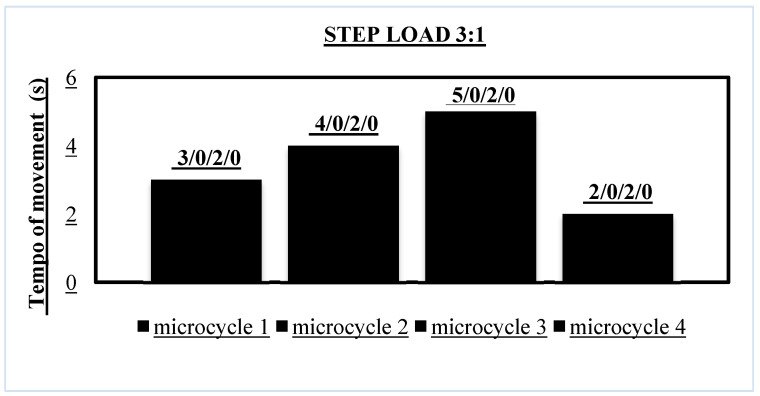
Tempo of movement (3/0/2/0) eccentric/isometric/concentric/isometric phases of each repetition/v-volitional tempo.

The rehabilitation protocol was developed using a 3:1 step load progression (Figure 4).

The protocol periodisation was based on unilateral (UNI) and bilateral (BIL) exercises (Table 2).

The progression and the load–deload phase were based on the step load method, where the exercise character was controlled and the movement tempo and TUT were controlled (Table 3, Table 4, Table 5 and Table 6) [32,33].

The judo athlete was included in 4 mesocycles of the rehabilitation protocol with the athlete in normobaric hypoxia with FiO_2_ = 15%, engaging mainly the lower muscle groups. Mesocycles consisted of 4 microcycles (1 microcycle = 7 days including 3 training sessions), which took place in the afternoon from 4:00 to 6:00 p.m. Resistance training took place on Mondays and Fridays, and running training on Wednesdays (Table 7 and Table 8).

First, immediately before the warm-up, the subject had to stay in the hypoxia chamber for 15 min. The next phase was a warm-up consisting of a 5 min walk on a mechanical treadmill and 5 min of cycling on a stationary bike. The last stage of the warm-up was to perform several exercises for the upper and lower body. Additionally, the judoka was regularly checked for oxygen saturation (Table 9).

## 3. Results

Results of GH, IGF-1, EPO hormone concentrations (baseline/after 2 mesocycles/after 4 mesocycles/after 48h) at different phases of each tempo of movement (Table 10).

Results before/after of peak torque extensors (Table 11).

Results before/after of knee extensor deficit (Table 12).

Results before/after of peak torque flexors (Table 13).

Results before/after of knee flexors deficit (Table 14).

Body composition results before/after rehabilitation protocol (Table 15).

Segment analysis results before/after rehabilitation protocol (Table 16).

Maximum oxygen uptake results before/after rehabilitation protocol (Table 17).

## 4. Discussion

The use of a hypoxic environment in training, taking into account the process of rehabilitation, tissue healing, the equalisation of muscle asymmetry, and increasing muscle hypertrophy, poses a number of questions to doctors, physiotherapists, and trainers because periodisation is a very holistic concept and is based on the use of many variables. It should also be noted that the limitation of this work is the use of a case study.

### 4.1. The Influence of Tempo of Movement and TUT on GH and IGF Concentrations

The intervention showed that the greatest increase in GH concentration was observed in the second and fourth mesocycles, falling in the third mesocycle based on the tempo of movement of 5/0/2/0 (Table 10). The GH concentration increased from the base value of 2.7 (ng/mL) to 6.7 times more in the second mesocycle at 18.4 (ng/mL) and to 7.5 times more in the fourth mesocycle, reaching a concentration of 20.3 (ng/mL). Very similar results were obtained in the study in [11], which concerned the preoperative ACL rehabilitation of a handball player in hypoxic conditions, and which also showed, in a similar protocol, the greatest increase in GH concentration in the phase preceding deload, reaching a peak concentration 10.81 times higher than the base value. We also observed a similar trend associated with an increase in GH concentration with an increase in the eccentric phase, which affects the total TUT volume of individual microcycles. Unfortunately, hormone sampling was limited to BIL training only, which is a limitation of this pilot study. Hence, the question arises whether the increase in TUT volume in the form of UNI would not cause an even greater increase in GH concentration, where the use of this form significantly affects the total TUT (Table 4 and Table 5). This raises another question: up to what point should the eccentric phase be extended in the tempo of movement, as this will undoubtedly influence the selection of the external load, which with such a number of repetitions will have a greater impact on sarcoplasmic hypertrophy, where, together with the hypoxic environment, it will shape strength endurance to a greater extent [34,35]. In several works, periodisation stages have generally been indicated with respect to what type of training should be applied to patients depending on the time since surgery, where in the fourth week after ACL surgery, the focus should be on muscle endurance [16]. Then, the hypertrophy stage should start from about the eighth week after surgery, because the initial muscle hypertrophy results from mechanisms related to neuromuscular adaptation and not hypertrophy itself, which would be in some sense consistent with our protocol. However, considering the structure of our periodisation, there is no muscle strength training, which should be implemented between 12 and 16 immediately before muscle power training, which also does not occur in our periodisation [36]. Therefore, when looking for a connection between the type of strength training and the level of GH, it is worth referring to the work of [25], analysing the effect of a tempo of movement of 2/0/2/0 and 5/0/3/0 on GH concentration in the barbell squat with 80% 1RM/five sets/3 min rests and repetitions to muscle failure. It indicated that the greatest increase in GH was achieved at the tempo of movement of 2/0/2/0 (13.7 ± 9.2 GH). In turn, at the tempo of 5/0/3/0, it increased on average by (9.95 ± 7.3 GH). The authors noted a higher average number of repetitions needed to achieve muscle failure at the tempo of movement of 2/0/2/0 (59.4 ± 5.5) and 5/0/3/0 (42.4 ± 5.4), which may have an impact on GH concentration. This would be consistent with the work in [37], where the highest GH and IGF-1 concentration was found with an increased number of series. It would also be worthwhile to extend the research to include the level of lactate concentration, as one of the studies comparing resistance training in two environments of hypoxia and normoxia, based on two exercises, using 50% 1RM, five sets, and 14 repetitions, showed a higher concentration of GH secretion. This suggests that the pituitary gland may be stimulated by increased metabolic accumulation (lactate and hydrogen ion concentration) in hypoxia compared to the normoxic group [25]. Therefore, the appropriate control of training measures (volume and intensity) through the use of periodisation in combination with the hypoxic environment may enhance this effect. This would be consistent with most scientific reports, where it has been proven that low intensity and high volume result in an increase in GH concentration in hypoxia [11,25,26,27,38]. Therefore, this was one of the factors that influenced the selection of measures for our rehabilitation protocol.

The results obtained regarding IGF-1 concentration during the second and fourth mesocycles conducted indicate that there is a neuroendocrine IGF-1 response after resistance training performed in hypoxic conditions (Table 10), which would be consistent with the work in [11,24], where it was shown that 6 weeks of resistance training in hypoxic conditions indicates a greater increase in IGF-1 concentration compared to group training under normoxic conditions. However, from the results obtained in our pilot study, we can see a certain two-track trend in IGF-1 concentration. First, by analysing the base values with the second mesocycle, we noticed a slight increase in IGF-1 concentration in each subsequent microcycle, reaching a peak in this mesocycle in the third microcycle (5/0/2/0) and in turn, the fourth microcycle (2/0/2/0) showed a decrease in IGF-1 concentration (Table 10). However, if we analyse the concentration of IGF-1 during the entire protocol, it is in the fourth mesocycle that we obtain the highest concentration but at the shortest tempo of movement (2/0/2/0). It should be mentioned that, in relation to the high fluctuations in GH concentration during the protocol, IGF-1 increases to a maximum of 226 (ng/mL) from the base value of 187 (ng/mL) by 37 (ng/mL), and at 48 h, its concentration is 224 (ng/mL). Therefore, the question arises whether the increase in IGF-1 may result from the character of training means such as the tempo of movement and TUT, which affect the volume and intensity of training (Table 6), or maybe the type of exercise (Table 6), which would not explain the IGF-1 concentration 48 h after the last training session.

### 4.2. Effect of Hypoxia and Rest Break on GH and IGF-1

If we want to implicate periodisation in rehabilitation protocols and their impact on GH and IGF-1, in addition to variables such as TUT and the tempo of movement, attention should also be paid to the impact of rest breaks between sets, where a large volume together with a hypoxic environment can lead to acute physiological reactions and thus affect the level of GH and IGF-1 [39]. Therefore, we decided to use a short rest break between sets, which was 80 s (Table 3). This has also been demonstrated in several works where pauses of 60–90 s were used. When selecting the altitude, we were guided by studies in which an increase in GH and IGF was observed from an altitude of 3000 m above sea level (FiO_2_ = 15%) [11,40].

### 4.3. Summary of the Effects on GH and IGF

The level of GH and IGF-1 was influenced by several factors. Undoubtedly, the hypoxic environment used (FiO_2_ = 15%) significantly increased metabolic stress, which caused the highest increase in GH. Another factor is the control of TUT, where the larger the volume of TUT in the training session (Table 4 and Table 5) based on the eccentric phase and the effort close to muscle failure based on the RIR scale (Table 3), the higher were the GH and IGF levels. This effect was enhanced by the use of short partial rest breaks between sets (80 s) in combination with six exercises in four sets and the hypoxic environment (FiO_2_ = 15%) significantly increased metabolic stress, which caused the highest increase in GH and IGF in the main accumulation phase per three microcycles (Table 10). This is consistent with the previous work [11], where this periodisation strategy showed the greatest increase in GH concentration in the accumulation phase in a similar overtreatment protocol in hypoxia. However, the results obtained as described above are not uniform. It can be clearly seen that the increase in GH and IGF-1 has an impact on the analysis of body composition (Table 15 and Table 16). This would be consistent with the work in [11,24,41], where it was shown that resistance training plus a hypoxic environment can affect hormone concentrations in a paracrine/autocrine manner. Hence, they can also exert an anabolic effect on bone by increasing osteoblast cell proliferation and bone mineral density as well as on skeletal muscle, thus helping to maintain lean body mass [26,41,42].

However, it is also necessary to note certain shortcomings of this pilot because the intake was performed only in BIL trainings where the overall TUT is much smaller in relation to UNI training. Therefore, extending the research to both forms of UNI and BIL training would allow for a comparison of both forms. Additionally, taking into account such variables as the artificial environment plus the entire periodisation of the microcycle, it should be noted that it is more targeted at athletes, because each series is close to muscle failure, which in combination with a short break intensifies the difficulty of this protocol.

### 4.4. EPO Concentration

At the very beginning, it is worth mentioning that EPO has been quite well studied under hypoxic conditions [43,44]. During the entire intervention, we observed a stepwise increase in EPO concentration reaching a peak value three times higher, at 16.2 (mlU/mL), than the base value of 5.2 (mlU/mL) at the end of the fourth mesocycle and 48 h after the last training session (Table 10), which was confirmed in the work in [11]. It is believed that EPO release is related to two factors: FiO_2_ and volume duration [45]. In our study, we used an altitude corresponding to 3000 m (FiO_2_ 15). This would be in line with the work [46], which recommends continuous exposure at 3000 m (FIO_2_ 15) for at least 114 min and in the case of 4000 m (FIO_2_ 13), for 84 min, which results in an increase in EPO. In our protocol, the “total time in hypoxia” was at least 105.5 min and at most 136.1 min (Table 6) depending on the form of UNI BIL training and the type of microcycle (Figure 4). It should also be taken into account that the EPO concentration was only measured immediately after each mesocycle, which fell on the supercompensation phase in the form of BIL, i.e., the shortest total training volume.

### 4.5. ACL Periodisation Process in Hypoxia

The use of periodisation in rehabilitation protocols, especially among athletes, is a key element that allows for the optimization of the entire process. Where individualization allows, depending on the stage of rehabilitation, the load can be controlled, with a controlled increase in relation to adaptive changes, taking into account the physiological and tolerance capabilities of the person undergoing rehabilitation, and taking into account the type of injury. By using such variable external load, number of repetitions, series, movement tempo, TUT, and rest breaks in the periodisation of rehabilitation protocols, we can consciously and purposefully control the rehabilitation or training process. However, the use of UNI and BIL exercises is one of the basic variables in the modification and intensification of individual training micro- and mesocycles [46]. The load progression method itself (step load 3:1) has already been described earlier. It is worth mentioning that, to our knowledge, this is the first work using load progression (step load 3:1) in postoperative ACL rehabilitation. Promising results from a pilot study [11] on pre-operative rehabilitation prompted us to use this periodisation method. It is also worth choosing the type of resistance exercises appropriately, because we must remember that the rehabilitation process is not only about increasing maximum strength, but is one of many variables influencing the effectiveness of the protocol.

The results show that the applied protocol in hypoxia significantly improved the peak torque of both knee extensors and flexors. Additionally, the deficit between the limbs was reduced (Table 11, Table 12, Table 13 and Table 14). That is why we used the UNI and BIL form of exercises in our protocol, which allows for the induction of many changes in a holistic approach to the entire rehabilitation process. First, the structure and nature of the exercise pattern, as well as the force with which it affects the ACL graft during specific exercises, are taken into account [47]. Another aspect is the reduction of swelling and the development of the quadriceps femoris muscle strength. Also important, as suggested by many authors, is the selection of rehabilitation exercises and progression in relation to the graft collection site [47,48].

The use of a hypoxic environment in muscle strength training, taking into account the process of rehabilitation, tissue healing, the equalization of muscle asymmetry, and increasing muscle hypertrophy, poses a number of questions to doctors, physiotherapists, and trainers because periodisation is a very holistic concept and is based on the use of many variables. From a review of contemporary literature on resistance training under artificial normobaric hypoxia conditions, we note a promising training optimization for increasing muscle strength and power. According to many authors, it is believed that the main mechanisms responsible for increased metabolic stress result from hypoxia, which is related to the effects of anaerobic exercise at low FiO_2_%. The use of the intensification of this process through moderate-intensity endurance training in hypoxic conditions increases the mechanism of metabolic stress induced by exercise (anabolic hormones, cytokines, reactive oxygen species, and oxidative stress factor), which affect positive changes in the formation of muscle hypertrophy [49,50,51].

To understand the use of both forms of exercise, it is necessary to consider the differences between them. The usefulness of BIL exercises, especially such as squats and deadlifts in training units, is their biomechanical structure, which allows for the use of increased total external load. Moreover, several studies have shown a correlation between such exercises and forms of activity such as jumping and sprinting over short distances [52,53,54]. There is also an increasing use of UNI exercises not only as complementary exercises, but as priority exercises in a given training session. This is due to the fact that in some activities, e.g., jumping, sprinting, and changes of direction, unilateral movements of individual body parts dominate [55]. In judo, these will include all attacks performed in the UNI form, e.g., Uchimata, Hani Goshi, and defensive activities, where the athlete must sometimes be able to keep individual body segments in balance, while outweighing the opponent’s muscle strength. This would confirm that resistance training based on UNI patterns is becoming increasingly popular among athletes, for whom the ability to transfer muscle force and power to a single muscle plays a key role in activities such as changing direction, deceleration, stabilization, etc. Therefore, UNI training seems to be an important factor, and research supports the thesis that unilateral training is essential during the periodisation of resistance training. What also differentiates UNI exercises from BIL is the phenomenon of bilateral deficit (BLD) and the selective recruitment of motor units [56]. The BLD phenomenon also consists of the fact that the maximum muscle strength of each limb is greater than the maximum muscle strength, where the BIL form is used [57,58]. Additionally, it is of great importance in the context of eliminating muscle asymmetry between limbs during rehabilitation proceedings. UNI exercises cause greater neuromuscular overload; furthermore, using appropriate exercise patterns in this form can stimulate the reconstruction of the range of motion of the operated limb. This process will depend on the inflammation of the limb, the applied tempo of movement, external load, and experience in resistance training [59,60,61,62]. Another advantage of using UNI exercises is the possibility of overloading the target muscle group while reducing the total load, and in our protocol, this was also enhanced by the hypoxic environment and the extended eccentric phase. This allows, for example, for the targeted stimulation of the quadriceps muscle with UNI exercise forms. The last indisputable advantage of UNI exercises is immediate feedback for the person leading the injured in the context of direct subjective information regarding limb symmetry.

### 4.6. VO_2_max

The protocol also consisted of running training in hypoxic conditions, which were also intended to stimulate the cardiorespiratory functions of the rehabilitated athlete. Currently, the use of a hypoxic environment in simulating cardiorespiratory functions is quite well known. It is recommended to use altitude (2000–3000 m), which results in improved oxygen transport due to a more effective secretion of erythropoietin and increased haemoglobin mass, which leads to increased VO_2_max. Positive adaptive changes are already noticeable after 2–3 weeks [63]. In one of the works, the authors, looking for factors influencing the increase in VO_2_max in conditions of intermittent hypoxia, state that apart from changes in blood concentration, an additional aspect is related to non-haematological mechanisms [64]. When looking for the most favourable percentage load of VO_2_max, a review of contemporary literature shows that apart from good intensity (moderate/high), the total volume also plays an important role both during one training session and micro- and mesocycles, where positive changes are visible after about (3–4 weeks) [65]. It should be noted that many authors state that it is the intensity of exercise that is the main factor influencing VO_2_max in conditions of hypoxia. Following this line of thought, several works state that moderate intensity close to the anaerobic threshold is more effective than higher intensity effort (close to or equal to maximum aerobic capacity) [65,66,67]. It is also worth mentioning that several studies indicate that intensity below 80% of VO2max does not induce positive changes [68,69]. Therefore, during one of the interventions in hypoxia conditions, it was shown that three microcycles based on 90% of VO_2_max intensity, using four series with a volume of 4/5 min are an effective means of improving aerobic capacity, which was confirmed during the ramp test [44]. Another study comparing four different VO_2_max intensities (long slow run at 60% VO_2_max, lactate threshold run at 80% VO_2_max, 15/15 interval training at 87.5% VO_2_max, and 4 × 4 min interval training at 87.5% VO_2_max) over eight microcycles (three training sessions per microcycle) showed very similar VO_2_max results. However, the greatest improvement in VO_2_max was observed after 4 × 4 min interval running (4 min run at 87.5% VO_2_max, followed by 3 min active recovery, and a jog at 60% VO_2_max) [65]. Therefore, the IHT method was used in our protocol. The patient completed a total of 16 running training sessions. With the patient’s safety in mind, we used a method of performing six 5 min intervals at a load of 70–80% Vo_2_max with 3–4 min breaks between each attempt so that the volume was not too high and the intensity resulting from the % VO_2_max as well as the hypoxic environment did not lead to excessive load on the operated limb, also taking into account other rehabilitation units. Additionally, the selection of training methods and means was very closely related to the current condition of the rehabilitated knee joint based on inflammation, excessive limping, and the patient’s subjective assessment. Therefore, participation in each training session involved consultation with a physiotherapist and a strength and conditioning coach. During 16 running sessions, the rehabilitated judoka achieved a significant increase in individual variables in the running test (Table 8). VO_2_max (ml/kg/min) increased from 54.2 to 65.8 (Table 17). Additionally, we saw a significant improvement in ΔLA 12′ res (mmol/L) at the point of the athlete’s introduction to full training/competition loads, which seems to be very important information, because judo is a discipline in which competition takes place in the form of a tournament system, hence the better lactate buffering significantly increases the feint’s capabilities (Table 17). The patient exercised depending on his disposition in the zone of 70–80% Hr max or speed km/h. In the initial period of rehabilitation, the main factor was the patient’s heart rate, while in the remaining three mesocycles, the main factor influencing the load was the running pace. Taking into account the patient’s safety and the adaptation to the load, we had to shorten the rest break in each subsequent mesocycle and increase the series to avoid maladaptation in the patient, which from our point of view, seemed to be a good balance between the intensity and volume of running sessions.

## 5. Conclusions

The periodisation of ACL rehabilitation in hypoxia based on a case study showed that the extension of the eccentric phase has a greater effect on GH concentration and a smaller effect on IGF-1 concentration, where after 48 h the GH concentration practically drops to the initial value, and IGF-1 remains at a high level. However, it is necessary to emphasize a certain limitation of the study, because the measurement of hormone concentration was performed only in the form of BIL training; extending the study to include concentrations in UNI training, where TUT is much higher, would allow for a better understanding of the kinematics of these hormones. It is worth mentioning that in our opinion, the use of such a protocol in a hypoxic environment based on periodisation using the tempo of movement, TUT, short rest breaks, and a high RIR scale suggests that this protocol is suitable for advanced athletes. This also directly translated into the reduction of the peak torque muscle asymmetry between the lower limbs of the knee extensors and flexors as well as the improvement of this parameter. Additionally, the use of the protocol in a hypoxic environment also affects VO_2_max, which is especially important in disciplines where circulatory and respiratory capabilities are also important in the context of the athlete’s return to full training loads. Thanks to load progression periodisation based on a step load of 3:1 among rehabilitated patients, we were able to induce targeted stimulation and, on the other hand, we avoided critical overload changes that, if uncontrolled, can extend the rehabilitation process.

Conducting a study on a larger population with the division of the applied periodisation into the hypoxic environment, normoxic environment, and control group would allow for a better understanding of the applied method. In addition, it seems necessary to include in future works an analysis of lactate concentration and hormones in UNI and BIL exercises.

## Figures and Tables

**Table 1:** Progressive exercise test.

STAGE	Elevation (%)	SPEED (km/h)	Duration (min)
1	0	6	3
2	0	8	3
3	0	10	3
4	2.5	12	3
5	5	12	3
6	7.5	12	3
8	10	12	3
9	12.5	12	3
10	15	12	3

**Table 2:** Exercise type.

M	I		II		III		IV	
UNI/BIL	UNI	BIL	UNI	BIL	UNI	BIL	UNI	BIL
1	Dumbbell split squat	Dumbbell squat	Goblet split squat dumbbell	Dumbbell goblet squat	Bulgarian split squat	Back squat low bar	Weighted pistol squats	Front squat
2	Seated band leg curl	Stiff leg deadlift	Single-leg trap bar RDL	Trap bar deadlift	Single-leg deadlift	Deadlift	Dumbbell single-leg jump squat	Dumbbell jump squat
3	Dumbbell single-arm chest press	Bench press	Seated single-arm overhead dumbbell press	Seated overhead dumbbell press	Swiss ball dumbbell single arm chest press	Swiss ball dumbbell chest press	Hollow- body floor single-arm press	Hollow- body floor Press
4	Single-leg glute bridge	Glute bridge	Single-leg hip thrust	Hip thrust	Supported step-up	Dumbbell goblet squat	Single-leg elevated hip thrust jump	Elevated hip thrust jump
5	Single-arm towel-grip landmine row	Towel-grip landmine row	Single-arm face pull	Dumbbell face pull	Single-arm towel-grip landmine row	Towel-grip landmine row	Swiss ball single leg curl	Swiss ball leg curl
6	Single-leg press	Leg press	Landmine single-arm thruster	Landmine thruster	Single-leg press	Leg press	Landmine single arm thruster	Landmine thruster

UNI—unilateral and BIL—bilateral.

**Table 3:** Resistance training variables.

Resistance Training Variables		
Variables	Unilateral	Bilateral
RIR	2–3	2–3
Sets (n)	4	4
Rest between sets (s)	80	80
Rest between exercises (s)	180	180
Reps (n)	16 (8 per side)	12
Number of exercises (n)	6	6

RIR—Reps in reserve.

**Table 4:** TUT of the UNI microcycles.

Time Under Tension					
Microcycle Tempo	TUT Exercise (s)			Total TUT Training Session (s)	Total TUT Training Session (min)
	TUT per Limb in a Series	TUT on Both Limbs in a Series	Total TUT in the Exercise		
3/0/2/0	40	80	360	2160	36
4/0/2/0	48	96	384	2304	38.4
5/0/2/0	56	112	448	2688	44.8
2/0/2/0	32	64	256	1536	25.6

TUT—time under tension.

**Table 5:** TUT of the BIL microcycles.

Microcycle Tempo	TUT Exercise (s)		Total TUT Training Session (s)	Total TUT Training Session (min)
	TUT on Both Limbs in a Series	Total TUT in the Exercise		
3/0/2/0	60	240	1440	24
4/0/2/0	72	288	1368	22.8
5/0/2/0	84	336	2016	33.6
2/0/2/0	48	192	1152	19.2

TUT—time under tension.

**Table 6:** Total time spent in hypoxia.

Action	Duration/Time of Training in Hypoxia (min)			
	Mesocycle			
	1 Microcycle	2 Microcycle	3 Microcycle	4 Microcycle
Staying Passive	15	15	15	15
Warm-up	15	15	15	15
Total TUT Training Session UNI/BIL	36/24	38.4/22.8	44.8/33.6	25.6/19.2
Break between sets	5.3	5.3	5.3	5.3
Break between exercises	21	21	21	21
Running with a break (+/−)	30	30	30	30
Staying passive	5	5	5	5
Total time in hypoxia UNI	127.3	129.7	136.1	116.9
Total time in hypoxia BIL	115.3	114.1	124.9	100.5

UNI—unilateral, BIL—bilateral, and TUT—time under tension.

**Table 7:** Microcycle variables.

Microcycle	1		2		3		4	
Training days	M 16–18 PM	U	M 16–18 PM	U	M 16–18 PM	U	M 16–18 PM	U
	W16–18 PM	R	W16–18 PM	R	W16–18 PM	R	W16–18 PM	R
	F 16–18 PM	B	F 16–18 PM	B	F 16–18 PM	B	F 16–18 PM	B

M—Monday, W—Wednesday, F—Friday, R—(running and bike), U—Unilateral, B—Bilateral.

**Table 8:** Running sessions.

Mesocycle	1	2	3	4
Training	4 × 1 km R/180s B HR zone—159–169 Tempo—05:25–04:40 min/km	4 × 1 km R/180s B HR zone—159–169 Tempo—05:25–04:40 min/km	4 × 1 km R/180s B HR zone—159–169 Tempo—05:25–04:40 min/km	4 × 1 km R/180s B HR zone—159 -169 Tempo—05:25–04:40 min/km

HR—heart rate, R—running, and B—break between running sets.

**Table 9:** Saturation measurements.

Measurement	SpO_2_ (% +/−)
Before warm-up	95–93
After warm-up	91–87
After each exercise	89–82

SpO_2_—saturation.

**Table 10:** Hormone concentrations results.

Variables	Baseline	After 2st Mesocycle				After 4st Mesocycle				48 h After
		Tempo 3/0/2/0	Tempo 4/0/2/0	Tempo 5/0/2/0	Tempo 2/0/2/0	Tempo 3/0/2/0	Tempo 4/0/2/0	Tempo 5/0/2/0	Tempo 2/0/2/0	
GH	2.7	16.3	18.2	18.4	17.1	17.2	17.6	20.3	17	2.8
IGF-1	187	197	208	210	207	206	221	220	226	220
EPO	5.2	-	-	-	10.4	-	-	-	16.2	16.1

GH—growth hormone, IGF-1—insulin growth factor, and EPO—erythropoietin.

**Table 11:** Peak torque extensors.

EXTENSORS						
Variables	Operated Limb			Healthy Limb		
	Before	After	Growth %	Before	After	Growth %
Peak Torque 60′/s—(Nm)	223	266	16.16	268	275	2.6
Peak Torque 120′/s—(Nm)	182	207	13.73	202	210	3.9
Peak Torque 180′/s—(Nm)	141	162	14.89	150	168	12

**Table 12:** Deficit in extensors.

Variables	Deficit (%)	
	Before	After
Peak torque 60′/s—(Nm/FFM)	16.79	3.38
Peak torque 120′/s—(Nm/FFM)	10.98	1.44
Peak Torque 180′/s—(Nm/FFM)	6.38	3.7

**Table 13:** Peak torque flexors.

FLEXORS						
Variables	Operated Limb			Healthy Limb		
	Before	After	Growth %	Before	After	Growth %
Peak Torque 60′/s—(Nm)	137	167	21.89	157	162	3.1
Peak Torque 120′/s—(Nm)	104	139	33.6	135	138	2.2
Peak Torque 180′/s—(Nm)	91	121	32.9	105	118	12.3

**Table 14:** Deficit in flexors.

Variables	Deficit (%)	
	Before	After
Peak torque 60′/s—(Nm/FFM)	14.59	5.98
Peak torque 120′/s—(Nm/FFM)	29.8	0.71
Peak Torque 180′/s—(Nm/FFM)	15.38	2.47

**Table 15:** Body composition.

Analysis	Before	After
Body height (cm)	178	178
Body weight (kg)	82.5	76.2
Bone mineral density (g/cm^3^)	1377	1377
Soft tissue (kg)	6.7	7
Fat tissue (kg)	11	7
Muscle mass (kg)	67.6	68

**Table 16:** Segment analysis.

Region	Fat Mass (%)		Total Mass (kg)		Fat Mass (g)		Muscle Mass (g)		Bone Mineral Content (g)	
	Before	After	Before	After	Before	After	Before	After	Before	After
Right arm	8.5	6	6.3	5.1	500	261	5449	6012	347	348
Left arm	10.5	5.8	6.3	5	624	244	5321	5936	341	341
Operated limb	12.5	8	13.9	6.9	1652	981	11,591	13,241	684	698
Healthy limb	11.8	7.6	13.8	6.7	1551	1001	11,549	13,030	671	686
Torso	16	9.4	16	13.2	11,015	8638	30,374	30,982	1183	1191

**Table 17:** VO_2_max—maximum oxygen uptake.

Variables	Before	After
Final load (km/h)/Elevation (%)/Time (s)	12/10/120	12/15/90
Load at the LT threshold (km/h)	10	12
VO_2_max (L/min)	4.55	5.09
VO_2_max (mL/kg/min)	54.2	65.8
VO_2_ at the LT threshold (mL/kg/min)	42.3	50.6
VEmax (L/min)	173.8	174.1
RERmax (VCO_2_/VO_2_)	1.23	1.2
HRmax (ud/min)	195	192
LAmax (mmol/L)	10.67	11.6
ΔLA 12′ res (mmol/L)	2.43	3.59

HRmax—maximal heart rat, VEmax—maximal ventilation, RER—respiratory exchange ratio, LA—lactate, ΔLA—maximal post exercise increase in lactate concentration, and VO_2_max—maximum oxygen uptake.

## Data Availability

The datasets generated and analysed during the current study are not publicly available but are available from the corresponding author who organized of the study.

## References

[1] Koshida S., Deguchi T., Miyashita K., Iwai K., Urabe Y. (2010). The Common Mechanisms of Anterior Cruciate Ligament Injuries in Judo: A Retrospective Analysis. Br. J. Sports Med..

[2] Degoutte F., Jouanel P., Filaire E. (2003). Energy Demands during a Judo Match and Recovery. Br. J. Sports Med..

[3] Franchini E., Nunes A.V., Moraes J.M., Del Vecchio F.B. (2007). Physical Fitness and Anthropometrical Profile of the Brazilian Male Judo Team. J. Physiol. Anthropol..

[4] Prados-Barbero F.J., Sánchez-Romero E.A., Cuenca-Zaldívar J.N., Selva-Sarzo F. (2024). Differences in Movement Patterns Related to Anterior Cruciate Ligament Injury Risk in Elite Judokas According to Sex: A Cross-Sectional Clinical Approach Study. Electron. J. Gen. Med..

[5] Akoto R., Lambert C., Balke M., Bouillon B., Frosch K.-H., Höher J. (2018). Epidemiology of Injuries in Judo: A Cross-Sectional Survey of Severe Injuries Based on Time Loss and Reduction in Sporting Level. Br. J. Sports Med..

[6] Kujala U.M., Taimela S., Antti-Poika I., Orava S., Tuominen R., Myllynen P. (1995). Acute Injuries in Soccer, Ice Hockey, Volleyball, Basketball, Judo, and Karate: Analysis of National Registry Data. BMJ.

[7] Malliaropoulos N.G., Callan M., Johnson J. (2014). Comprehensive Training Programme for Judo Players Nine plus 9+: Possible Lower Limb Primary Injury Prevention. Muscles Ligaments Tendons J..

[8] Majewski M., Susanne H., Klaus S. (2006). Epidemiology of Athletic Knee Injuries: A 10-Year Study. Knee.

[9] Takahashi S., Nagano Y., Ito W., Kido Y., Okuwaki T. (2019). A Retrospective Study of Mechanisms of Anterior Cruciate Ligament Injuries in High School Basketball, Handball, Judo, Soccer, and Volleyball. Medicine.

[10] Kasahara, Martin D., Humberstone C., Yamamoto T., Nakamura T. (2015). Classification of Sports Injuries in Japanese University Judo Players and Analysis of Associated Physical Fitness Characteristics. J. Sci. Med. Sport.

[11] Motowidło J., Stronska-Garbien K., Bichowska-Pawęska M., Kostrzewa M., Zając A., Ficek K., Drozd M. (2024). Effect of Step Load Based on Time under Tension in Hypoxia on the ACL Pre-Operative Rehabilitation and Hormone Levels: A Case Study. J. Clin. Med..

[12] Amann M., Romer L.M., Subudhi A.W., Pegelow D.F., Dempsey J.A. (2007). Severity of Arterial Hypoxaemia Affects the Relative Contributions of Peripheral Muscle Fatigue to Exercise Performance in Healthy Humans. J. Physiol..

[13] Feriche B., García-Ramos A., Morales-Artacho A.J., Padial P. (2017). Resistance Training Using Different Hypoxic Training Strategies: A Basis for Hypertrophy and Muscle Power Development. Sports Med. Open.

[14] Scott B.R., Slattery K.M., Sculley D.V., Dascombe B.J. (2014). Hypoxia and Resistance Exercise: A Comparison of Localized and Systemic Methods. Sports Med..

[15] Lorenz D.S., Reiman M.P., Walker J.C. (2010). Periodization: Current Review and Suggested Implementation for Athletic Rehabilitation. Sports Health.

[16] Harries S.K., Lubans D.R., Callister R. (2015). Systematic Review and Meta-Analysis of Linear and Undulating Periodized Resistance Training Programs on Muscular Strength. J. Strength Cond. Res..

[17] Kraemer W.J., Ratamess N.A., Flanagan S.D., Shurley J.P., Todd J.S., Todd T.C. (2017). Understanding the Science of Resistance Training: An Evolutionary Perspective. Sports Med..

[18] Flagg K.Y., Karavatas S.G., Thompson S., Bennett C. (2019). Current Criteria for Return to Play after Anterior Cruciate Ligament Reconstruction: An Evidence-Based Literature Review. Ann. Transl. Med..

[19] Mayr H.O., Stoehr A., Herberger K.T., Haasters F., Bernstein A., Schmal H., Prall W.C. (2021). Histomorphological Alterations of Human Anterior Cruciate Ligament Grafts During Mid-Term and Long-Term Remodeling. Orthop. Surg..

[20] Hollings S., Hopkins W., Hume P. (2012). Environmental and Venue-Related Factors Affecting the Performance of Elite Male Track Athletes. Eur. J. Sport. Sci..

[21] Hamlin M.J., Hopkins W.G., Hollings S.C. (2015). Effects of Altitude on Performance of Elite Track-and-Field Athletes. Int. J. Sports Physiol. Perform..

[22] McLean B.D., Buttifant D., Gore C.J., White K., Liess C., Kemp J. (2013). Physiological and Performance Responses to a Preseason Altitude-Training Camp in Elite Team-Sport Athletes. Int. J. Sports Physiol. Perform..

[23] Ishii Y., Deie M., Adachi N., Yasunaga Y., Sharman P., Miyanaga Y., Ochi M. (2005). Hyperbaric Oxygen as an Adjuvant for Athletes. Sports Med..

[24] Chycki J., Czuba M., Gołaś A., Zając A., Fidos-Czuba O., Młynarz A., Smółka W. (2016). Neuroendocrine Responses and Body Composition Changes Following Resistance Training Under Normobaric Hypoxia. J. Hum. Kinet..

[25] Kon M., Ikeda T., Homma T., Akimoto T., Suzuki Y., Kawahara T. (2010). Effects of Acute Hypoxia on Metabolic and Hormonal Responses to Resistance Exercise. Med. Sci. Sports Exerc..

[26] Kon M., Ikeda T., Homma T., Suzuki Y. (2012). Effects of Low-Intensity Resistance Exercise under Acute Systemic Hypoxia on Hormonal Responses. J. Strength Cond. Res..

[27] Yan B., Lai X., Yi L., Wang Y., Hu Y. (2016). Effects of Five-Week Resistance Training in Hypoxia on Hormones and Muscle Strength. J. Strength Cond. Res..

[28] Mendias C.L., Enselman E.R.S., Olszewski A.M., Gumucio J.P., Edon D.L., Konnaris M.A., Carpenter J.E., Awan T.M., Jacobson J.A., Gagnier J.J. (2020). The Use of Recombinant Human Growth Hormone to Protect Against Muscle Weakness in Patients Undergoing Anterior Cruciate Ligament Reconstruction: A Pilot, Randomized Placebo-Controlled Trial. Am. J. Sports Med..

[29] Humac Norm Testing & Rehabilitation System. https://docplayer.net/37846364-Humac-norm-testing-rehabilitation-system.html.

[30] Almosnino S., Stevenson J.M., Bardana D.D., Diaconescu E.D., Dvir Z. (2012). Reproducibility of Isokinetic Knee Eccentric and Concentric Strength Indices in Asymptomatic Young Adults. Phys. Ther. Sport..

[31] Buttar K., Scholar, Saboo N., Kacker S. (2019). A Review: Maximal Oxygen Uptake (VO_2_ Max) and Its Estimation Methods. Int. J. Phys. Educ. Sports Health.

[32] de Paula Simola R.Á., Harms N., Raeder C., Kellmann M., Meyer T., Pfeiffer M., Ferrauti A. (2015). Assessment of Neuromuscular Function after Different Strength Training Protocols Using Tensiomyography. J. Strength Cond. Res..

[33] Kojić F., Ranisavljev I., Ćosić D., Popović D., Stojiljković S., Ilić V. (2021). Effects of Resistance Training on Hypertrophy, Strength and Tensiomyography Parameters of Elbow Flexors: Role of Eccentric Phase Duration. Biol. Sport..

[34] Roberts M.D., Haun C.T., Vann C.G., Osburn S.C., Young K.C. (2020). Sarcoplasmic Hypertrophy in Skeletal Muscle: A Scientific “Unicorn” or Resistance Training Adaptation?. Front. Physiol..

[35] Henselmans M., Schoenfeld B.J. (2014). The Effect of Inter-Set Rest Intervals on Resistance Exercise-Induced Muscle Hypertrophy. Sports Med..

[36] Kakavas G., Malliaropoulos N., Bikos G., Pruna R., Valle X., Tsaklis P., Maffulli N. (2020). Periodization in Anterior Cruciate Ligament Rehabilitation: A Novel Framework. Med. Princ. Pract..

[37] Wilk M., Golas A., Stastny P., Nawrocka M., Krzysztofik M., Zajac A. (2018). Does Tempo of Resistance Exercise Impact Training Volume?. J. Hum. Kinet..

[38] Kon M., Ohiwa N., Honda A., Matsubayashi T., Ikeda T., Akimoto T., Suzuki Y., Hirano Y., Russell A.P. (2014). Effects of Systemic Hypoxia on Human Muscular Adaptations to Resistance Exercise Training. Physiol. Rep..

[39] Scott B.R., Slattery K.M., Sculley D.V., Lockhart C., Dascombe B.J. (2017). Acute Physiological Responses to Moderate-Load Resistance Exercise in Hypoxia. J. Strength Cond. Res..

[40] Katayama K., Goto K., Ishida K., Ogita F. (2010). Substrate Utilization during Exercise and Recovery at Moderate Altitude. Metabolism.

[41] Guardado I., Ureña B., Cardenosa A., Cardenosa M., Camacho G., Andrada R. (2020). Effects of Strength Training under Hypoxic Conditions on Muscle Performance, Body Composition and Haematological Variables. Biol. Sport..

[42] Friedmann B., Kinscherf R., Borisch S., Richter G., Bärtsch P., Billeter R. (2003). Effects of Low-Resistance/High-Repetition Strength Training in Hypoxia on Muscle Structure and Gene Expression. Pflugers Arch..

[43] Semenza G.L. (2004). O2-Regulated Gene Expression: Transcriptional Control of Cardiorespiratory Physiology by HIF-1. J. Appl. Physiol..

[44] Czuba M., Zając A., Maszczyk A., Roczniok R., Poprzęcki S., Garbaciak W., Zając T. (2013). The Effects of High Intensity Interval Training in Normobaric Hypoxia on Aerobic Capacity in Basketball Players. J. Hum. Kinet..

[45] Mackenzie R.W.A., Watt P.W., Maxwell N.S. (2008). Acute Normobaric Hypoxia Stimulates Erythropoietin Release. High. Alt. Med. Biol..

[46] Liao K.-F., Nassis G.P., Bishop C., Yang W., Bian C., Li Y.-M. (2022). Effects of Unilateral vs. Bilateral Resistance Training Interventions on Measures of Strength, Jump, Linear and Change of Direction Speed: A Systematic Review and Meta-Analysis. Biol. Sport..

[47] Cavanaugh J.T., Powers M. (2017). ACL Rehabilitation Progression: Where Are We Now?. Curr. Rev. Musculoskelet. Med..

[48] Schwery N.A., Kiely M.T., Larson C.M., Wulf C.A., Heikes C.S., Hess R.W., Giveans M.R., Solie B.S., Doney C.P. (2022). Quadriceps Strength Following Anterior Cruciate Ligament Reconstruction: Normative Values Based on Sex, Graft Type and Meniscal Status at 3, 6 & 9 Months. Int. J. Sports Phys. Ther..

[49] Emery C.A., Roy T.-O., Whittaker J.L., Nettel-Aguirre A., van Mechelen W. (2015). Neuromuscular Training Injury Prevention Strategies in Youth Sport: A Systematic Review and Meta-Analysis. Br. J. Sports Med..

[50] Ebben W.P., Blackard D.O. (2001). Strength and Conditioning Practices of National Football League Strength and Conditioning Coaches. J. Strength Cond. Res..

[51] Ebert J.R., Edwards P.K., Fick D.P., Janes G.C. (2017). A Systematic Review of Rehabilitation Exercises to Progressively Load the Gluteus Medius. J. Sport. Rehabil..

[52] Chaouachi A., Brughelli M., Chamari K., Levin G.T., Ben Abdelkrim N., Laurencelle L., Castagna C. (2009). Lower Limb Maximal Dynamic Strength and Agility Determinants in Elite Basketball Players. J. Strength Cond. Res..

[53] Hornsby W.G., Gentles J.A., Haff G.G., Stone M.H., Buckner S.L., Dankel S.J., Bell Z.W., Abe T., Loenneke J.P. (2018). What Is the Impact of Muscle Hypertrophy on Strength and Sport Performance?. Strength Cond. J..

[54] Comfort P., Haigh A., Matthews M.J. (2012). Are Changes in Maximal Squat Strength during Preseason Training Reflected in Changes in Sprint Performance in Rugby League Players?. J. Strength Cond. Res..

[55] Papla M., Krzysztofik M., Wojdala G., Roczniok R., Oslizlo M., Golas A. (2020). Relationships between Linear Sprint, Lower-Body Power Output and Change of Direction Performance in Elite Soccer Players. Int. J. Environ. Res. Public. Health.

[56] Mullican K., Nijem R. (2016). Are Unilateral Exercises More Effective Than Bilateral Exercises?. Strength Cond. J..

[57] Whitcomb E., Ortiz O., Toner J., Kuruganti U. (2021). The Bilateral Limb Deficit (BLD) Phenomenon during Leg Press: A Preliminary Investigation into Central and Peripheral Factors. BMC Sports Sci. Med. Rehabil..

[58] Bračič M., Supej M., Peharec S., Bačič P., Čoh M. (2010). An Investigation of the Influence of Bilateral Deficit on the Counter-Movement Jump Performance in Elite Sprinters. Kinesiology.

[59] McCurdy K., O’Kelley E., Kutz M., Langford G., Ernest J., Torres M. (2010). Comparison of Lower Extremity EMG between the 2-Leg Squat and Modified Single-Leg Squat in Female Athletes. J. Sport. Rehabil..

[60] Chapman A.R., Vicenzino B., Blanch P., Hodges P.W. (2008). Patterns of Leg Muscle Recruitment Vary between Novice and Highly Trained Cyclists. J. Electromyogr. Kinesiol..

[61] Akima H., Kuno S., Takahashi H., Fukunaga T., Katsuta S. (2000). The Use of Magnetic Resonance Images to Investigate the Influence of Recruitment on the Relationship between Torque and Cross-Sectional Area in Human Muscle. Eur. J. Appl. Physiol..

[62] Gryzlo S.M., Patek R.M., Pink M., Perry J. (1994). Electromyographic Analysis of Knee Rehabilitation Exercises. J. Orthop. Sports Phys. Ther..

[63] Bunn H.F., Poyton R.O. (1996). Oxygen Sensing and Molecular Adaptation to Hypoxia. Physiol. Rev..

[64] Czuba M., Waskiewicz Z., Zajac A., Poprzecki S., Cholewa J., Roczniok R. (2011). The Effects of Intermittent Hypoxic Training on Aerobic Capacity and Endurance Performance in Cyclists. J. Sports Sci. Med..

[65] Helgerud J., Høydal K., Wang E., Karlsen T., Berg P., Bjerkaas M., Simonsen T., Helgesen C., Hjorth N., Bach R. (2007). Aerobic High-Intensity Intervals Improve VO_2_max More than Moderate Training. Med. Sci. Sports Exerc..

[66] Dufour S.P., Ponsot E., Zoll J., Doutreleau S., Lonsdorfer-Wolf E., Geny B., Lampert E., Flück M., Hoppeler H., Billat V. (2006). Exercise Training in Normobaric Hypoxia in Endurance Runners. I. Improvement in Aerobic Performance Capacity. J. Appl. Physiol..

[67] Roels B., Bentley D.J., Coste O., Mercier J., Millet G.P. (2007). Effects of Intermittent Hypoxic Training on Cycling Performance in Well-Trained Athletes. Eur. J. Appl. Physiol..

[68] Ventura N., Hoppeler H., Seiler R., Binggeli A., Mullis P., Vogt M. (2003). The Response of Trained Athletes to Six Weeks of Endurance Training in Hypoxia or Normoxia. Int. J. Sports Med..

[69] Truijens M.J., Toussaint H.M., Dow J., Levine B.D. (2003). Effect of High-Intensity Hypoxic Training on Sea-Level Swimming Performances. J. Appl. Physiol..

